# SATB1 is an independent prognostic factor in radically resected upper gastrointestinal tract adenocarcinoma

**DOI:** 10.1007/s00428-014-1667-6

**Published:** 2014-10-19

**Authors:** Charlotta Hedner, Alexander Gaber, Dejan Korkocic, Björn Nodin, Mathias Uhlén, Eugenia Kuteeva, Henrik Johannesson, Karin Jirström, Jakob Eberhard

**Affiliations:** 1 Department of Clinical Sciences, Division of Oncology and Pathology, Lund University, Skåne University Hospital, 221 85 Lund, Sweden; 2 Science for Life Laboratory, AlbaNova University Center, Royal Institute of Technology, 106 91 Stockholm, Sweden; 3 School of Biotechnology, AlbaNova University Center, Royal Institute of Technology, 106 91 Stockholm, Sweden; 4 Atlas Antibodies AB, AlbaNova University Center, 106 91 Stockholm, Sweden

**Keywords:** SATB1, SATB2, Gastric adenocarcinoma, Esophageal adenocarcinoma, Prognosis

## Abstract

Gastric cancer is the second most common cause of cancer-related death worldwide, and the incidence of esophageal adenocarcinoma is rising. While some progress has been made in treatment strategies, overall survival remains very poor for patients with adenocarcinoma in the upper gastrointestinal tract. Special AT-rich sequence binding protein 1 (SATB1) is a global genome organizer that has been demonstrated to promote aggressive tumor behavior in several different types of cancer, including gastric cancer. The prognostic value of SATB1 expression in esophageal cancer has, however, not yet been described. In this study, expression of SATB1 was examined by immunohistochemistry on tissue microarrays prepared from tissue samples from 175 patients with adenocarcinoma of the esophagus, cardia, or stomach and containing normal tissue, intestinal metaplasia, primary tumors, and metastases. A well-validated antibody was used. We found SATB1 to be an independent prognostic factor in patients with a radically resected tumor, correlating with shorter overall survival as well as with shorter recurrence-free survival. SATB1 expression was also found to be significantly lower in primary tumors associated with intestinal metaplasia than those without intestinal metaplasia. This observation is of potential biological interest as it has been proposed that intestinal metaplasia-associated tumors constitute a less aggressive phenotype.

**Electronic supplementary material:**

The online version of this article (doi:10.1007/s00428-014-1667-6) contains supplementary material, which is available to authorized users.

## Introduction

Gastric cancer was for a long time the leading cause of cancer-related death worldwide [1]. Due principally to better living conditions, the incidence rate has been declining, but gastric cancer is still the second most common cause of cancer-related death worldwide [1–4] with 5-year survival rates ranging from 10 to 27 % depending on the anatomical location and also on which part of the world the patient resides in [3]. In contrast, the incidence of esophageal adenocarcinomas has been steadily rising in the past decades [2, 5].

With such poor survival rates, there is an evident and immediate need to sharpen our diagnostic, prognostic, and treatment tools in order to improve survival rates for these patients. When it comes to treatment, there has been some progress during the last few years with large studies showing improved survival rates in patients receiving neoadjuvant or perioperative chemotheraphy and/or radiotherapy instead of surgical treatment only [6–8]. Although this is a step forward, there has been less progress in understanding the mechanisms that drive cancer progression and metastasis in these cancers and in the identification of clinically useful prognostic and treatment response predictive biomarkers. Hence, there is a need for novel biomarkers which might improve individualized treatment stratification and ultimately survival of patients with cancer in the upper gastrointestinal tract.

Special AT-rich sequence binding protein 1 (SATB1) is a global genome organizer [9] initially identified in thymocytes [10] and that recently attracted some attention as a putative cancer biomarker. Chromosomes are organized in the nucleus of a cell in such a way that only part of the genome is expressed [11]. This is regulated through chromatin proteins involved in chromatin compaction, which make chromatin fold into three-dimensional structures which in turn determine which genes might be transcribed [9]. The genome harbors regions characterized by DNA sequences with one strand having A’s, T’s, and C’s but no G’s (ATC sequences, also known as BURs) [9]. When the ATC sequence context is disrupted by mutations, SATB1 binding is abolished [10]. SATB1 binds specifically to these ATC sequences, resulting in chromatin folding into loop domains which enables regulation of the expression of multiple genes [9, 11]. Furthermore, SATB1 also provides a nuclear platform for docking of chromatin-remodeling enzymes, and through this mechanism, coordinates expression of several hundreds up to a thousand genes [9, 11, 12].

Expression of SATB1 has been correlated with a more aggressive tumor phenotype and worse prognosis in cancer of the breast [9, 12], ovary [13], colorectum [14–16], and larynx [17]. Han et al. suggested that its expression is necessary for breast cancer to become metastatic [12]. Other papers have reported contradicting results regarding the role of SATB1 in cancer progression in breast and colorectal cancer [18–20]. This may be due to differences in experimental design, e.g., examining SATB1 expression in total RNA transcripts from tumor tissue specimens as opposed to scoring SATB1 protein levels in individual tumor cells by immunohistochemistry [21]. In addition, differences in specificity of the antibodies used might significantly impact on the results.

SATB1 expression in gastric cancer has previously been examined in two studies on Chinese populations, both indicating that SATB1 expression is independently associated with worse prognosis [22, 23]. An in vitro study supported the correlation between SATB1 expression and aggressive tumor behavior and also suggested that SATB1 plays a role in multidrug resistance [24]. SATB1 expression has, to our best knowledge, not been examined in esophageal cancer. The aim of our study was to examine longitudinal expression of SATB1 and its prognostic significance in adenocarcinomas of the esophagus, cardia, and stomach.

## Materials and methods

### Study design and participants

The study was performed on a consecutive cohort of 175 patients with adenocarcinoma in the upper gastrointestinal tract (esophagus, cardia, and stomach) who had been surgically treated in the university hospitals of Lund and Malmö from January 1, 2006–December 31, 2010. The cohort has been described in detail previously [25, 26]. In brief, all tumors were histopathologically re-examined including confirmation of diagnosis, number of lymph nodes with metastasis (re-classified following the standardized TNM 7 classification by the American Joint Committee on Cancer (AJCC) [27]), and presence of intestinal metaplasia (Barrett’s esophagus or gastric intestinal metaplasia (IM)) with or without dysplasia.

Clinical data, information on recurrence and vital status, or cause of death were obtained from the medical charts. The mean follow-up time for patients alive was 5.2 years (range 2.7–7.7).

Patient and tumor characteristics are provided in Supplemental Table 1.

Approval was obtained from the ethics committee at Lund University (ref no. 445-07).

### Tissue microarrays

Tissue microarrays (TMAs) were constructed using a semi-automated arraying device (TMArrayer, Pathology Devices, Westminister, MD, USA) as previously described [25, 28]. Tissue was taken from viable, non-necrotic areas in duplicate 1-mm cores from primary tumors. In addition, lymph node metastases were sampled in 81 cases, IM (including Barrett’s esophagus) in 73 cases, normal squamous epithelium in 96 cases, and normal gastric mucosa in 131 cases. Duplicate cores were obtained from different blocks of the primary tumor and different lymph node metastases in cases with more than one metastasis. Normal squamous epithelium and gastric mucosa were represented in single cores and IM in 1–3 cores.

### Antibody validation—Western blot

Western blot analyses were performed according to standard protocols on SATB1 and SATB2 overexpression lysates co-expressed with a C-terminal myc-DDK tag (∼3.1 kDa) in mammalian HEK293T cells (LY427355 and LY414656, respectively, Origene Technologies, Rockville, MD, USA). Briefly, 2 μl of SATB1 and SATB2 overexpression lysate was separated on precast 4–20 % CriterionTGX SDS-PAGE gradient gels (Bio-Rad Laboratories, Hercules, CA) under reducing conditions, followed by blotting to PVDF membranes (Trans-Blot® Turbo™ Midi PVDF Transfer Packs, Bio-Rad Laboratories, Hercules, CA), according to the instructions of the manufacturer. Membranes were blocked for 45 min at RT in blocking buffer (5 % dry milk, 0.5 % Tween 20, 1× TBS) prior to addition of antibody (anti-SATB1, clone EPR3895, Epitomics, Burlingame, CA, USA; anti-SATB2 #AMAb90679 CL0320, Atlas Antibodies AB, Stockholm, Sweden; or anti-DDK Tag# TA50011, Origene Technologies, Rockville, MD, USA), diluted to a final concentration of 1 μg/ml in blocking buffer. Following incubation for 1 h with primary antibody, the membranes were washed 4 × 5 min in 1× TBS with 0.1 % Tween 20. Horseradish peroxidase (HRP)-conjugated secondary antibody (swine anti-rabbit antibody #P0399 or goat anti-mouse antibody #P0447, Dako), diluted 1:3,000 in blocking buffer, was added to the membranes and incubated for 30 min followed by a final round of washing. Detection was carried out using chemiluminescence HRP substrate (Immobilon, EMD Millipore Corporation, Billerica, MA, USA) according to the manufacturer’s instructions.

### Antibody validation—immunohistochemistry

The specificity of SATB1 and SATB2 antibodies was further evaluated in immunohistochemical experiments.

Tissue sections (4 μm) were cut from TMAs containing 18 normal (fallopian tube, cervix, endometrium, placenta, testis, prostate, liver, pancreas, rectum, colon, stomach, duodenum, small intestine, cerebellum, cerebral cortex, skin, skeletal muscle, and tonsil) and 7 cancer (prostate, colorectal, ventricular, renal, liver, lung, and breast) tissues. Prior to immunostaining, the sections were baked at 50 °C overnight and deparaffinized in xylene and graded ethanol. Antigen retrieval was then performed using citrate buffer pH 6 (ThermoFisher Scientific, Waltham, MA, USA) in decloaking chamber (Biocare Medical, Walnut Creek, CA, USA). Sections were stained with anti-SATB1rabbit monoclonal antibody (Clone EPR3895, Epitomics, Burlingame, CA, USA) diluted 1:100 or mouse monoclonal antibody against SATB2 (AMAb90679, CL0320, Atlas Antibodies, Stockholm, Sweden) diluted 1:1,000 in Autostainer 480S (ThermoFisher Scientific, Waltham, MA, USA) using a commercial kit (UltraVision LP HRP polymer®, Primary Antibody Enhancer, Ultra V Block and DAB plus substrate system®, ThermoFisher Scientific, Waltham, MA, USA). Slides were counterstained with hematoxylin and mounted using Pertex.

Slides were examined, and images were taken using an automated system (VSlide, Metasystems).

### Immunohistochemistry and staining evaluation

For immunohistochemistry, 4-μm TMA sections were baked in a heated chamber for 120 min at 60 °C. Antigen retrieval for Ki67, p53, and SATB1 was performed using HIER pH 9 (PT-link system Dako, Glostrup, Denmark), and for SATB2 pH 6 (decloaking chamber, Biocare Medical, Walnut Creek, CA, USA).

For Ki67, a monoclonal antibody (clone MIB1 Dako, diluted 1:50) was applied in a BenchMark ULTRA (Ventana Medical systems, Tuscon, AZ, USA).

Expression of p53 was analyzed using a monoclonal antibody (clone DO-7, Dako). Expression of SATB1 was assessed using a monoclonal antibody (Clone EPR 3895, Epitomics, Burlingame, CA, USA, diluted 1:100), as for SATB2 (AMAb90679 CLO320, Atlas Antibodies, diluted 1:1,000), and staining for all three antibodies was performed in an Autostainer Plus (Dako, Glostrup, Denmark). DAB was used as chromogen, and the slides were counterstained with hematoxylin.

For assessment of Ki67 expression, the fraction of Ki67 nuclear staining was categorized as follows: 0–1, 2–10, 11–20, 21–50, and >50 %. For statistical analysis, three categories were applied: 0–20, 21–50, and >50 %.

The fraction of p53 staining was categorized as follows: 0–1, 2–10, 11–50, and >50 %. For statistical analysis, three categories were applied: 0–1, 2–50, and >50 %.

The estimated fraction of cells with nuclear SATB1 expression was denoted and after that, transformed into five categories of 0 (0–1 %), 1 (2–25 %), 2 (26–50 %), 3 (51–75 %), and 4 (>75 %). The predominant nuclear intensity was estimated as negative (0), weak (1), moderate (2), or strong (3). For statistical analysis, a combined nuclear score was constructed by multiplying fraction and intensity, and any intensity of staining of ≥2 % of the cells was denoted as positive SATB1 staining. In line with previous studies, stromal lymphocytes served as a positive control for SATB1 [9]. Evaluation of nuclear SATB2 expression was recorded in the same manner as described for SATB1.

All stained sections were evaluated by two independent observers who were blinded to clinical and outcome data.

## Statistical analysis

The chi-squared test was applied to analyze the relationship between SATB1 expression and clinicopathological parameters. Overall survival (OS) rates and recurrence-free survival (RFS) time according to SATB1 negativity versus SATB1 positivity were calculated using Kaplan-Meier analysis. To assess differences in the Kaplan-Meier curves, the log-rank test was used. Unadjusted and adjusted hazard ratios (HR) for OS and RFS were calculated by Cox regression proportional hazard modeling. The adjusted model included age, sex, T stage, N stage, M stage, differentiation, and SATB1 expression.

For some subjects, information on one or several markers was not available. Missing values were coded as a separate category for categorical variables. Missing values for categorical variables co-varied. The adjusted model did not converge due to many constant values. In order to avoid this, only patients with information on SATB1 expression were included in the adjusted analysis.

A backward conditional method was used for variable selection in the adjusted model.

For all analyses, IBM SPSS Statistics version 20.0 (SPSS Inc., Chicago, IL, USA) was used. *p* values <0.05 were considered significant. All tests were two-sided.

## Results

### Antibody validation

**Fig. 1:**
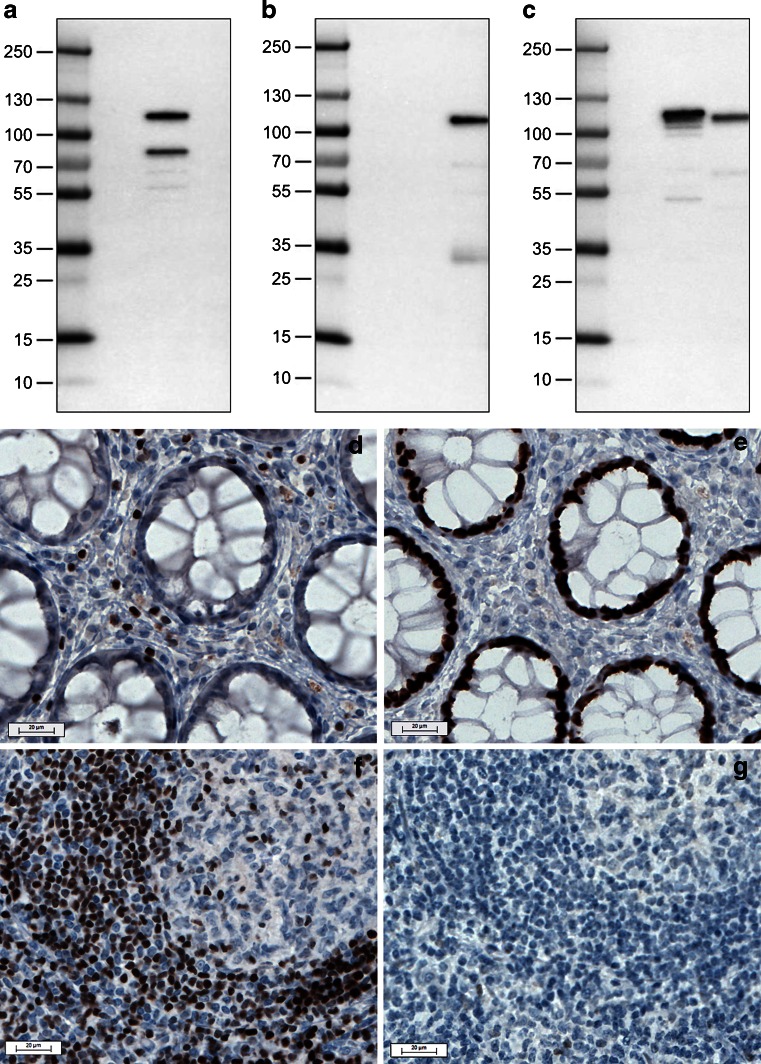
Assessment of specificity of anti-SATB1 and anti-SATB2 antibodies using Western blot (**a**–**c**) and immunohistochemistry (**d**–**e**) analyses. Western blot results following incubation with anti-SATB1 (**a**), anti-SATB2 (**b**), or anti-DDK Tag (**c**) antibodies (lane 1, molecular weight marker; lane 2, negative control lysate; lane 3, SATB1; and lane 4, SATB2-overexpressing mammalian HEK293T lysate). Note specific binding of antibodies to their respective lysates. Immunohistochemistry results following staining with anti-SATB1 (**d**, **f**) and anti-SATB2 (**e**, **g**) antibodies in rectum (**d**, **e**) and tonsil (**f**, **g**). Note strong nuclear immunoreactivity in a subset of lymphocytes following staining with anti-SATB1 antibody both in rectum and tonsil and absence of nuclear immunoreactivity in glandular epithelium of rectum. Staining with anti-SATB2 displays strong nuclear positivity in rectum glandular cells, while lymphoid cells are mainly negative

Western blot analyses were performed on HEK293T cell lysates overexpressing the full-length SATB1 and SATB2 proteins (Fig. 1a–c) and revealed that both antibodies bind specifically and selectively to their respective target protein.

Different staining patterns for SATB1 and SATB2 were obtained on normal and cancer tissues. SATB1 immunoreactivity was limited to a subpopulation of lymphoid cells in various tissues (Fig. 1d, f), but no immunoreactivity was observed in glandular cells in the rectum (Fig. 1d), colon, or in colorectal cancer (data not shown). In addition, weak to moderate nuclear staining was seen in single cells in the fallopian tube, seminiferous tubules, and in the majority of glandular cells in prostate. Strong nuclear immunoreactivity was detected in single neurons in cerebral cortex.

Very strong immunoreactivity for SATB2 was observed in colorectal mucosa (Fig. 1e) as well as in colorectal cancer (data not shown). Moderate nuclear positivity was seen in a subset of neurons in cerebral cortex and single glandular cells in the duodenum, kidney, and prostate. In tonsil, only single lymphoid cells displayed very weak nuclear immunoreactivity (Fig. 1g).

Taken together, IHC and Western blot validation demonstrates that the two antibodies used in this study are highly specific to their respective target protein, despite extensive sequence similarity of the two proteins.

### Longitudinal SATB1 expression

**Fig. 2:**
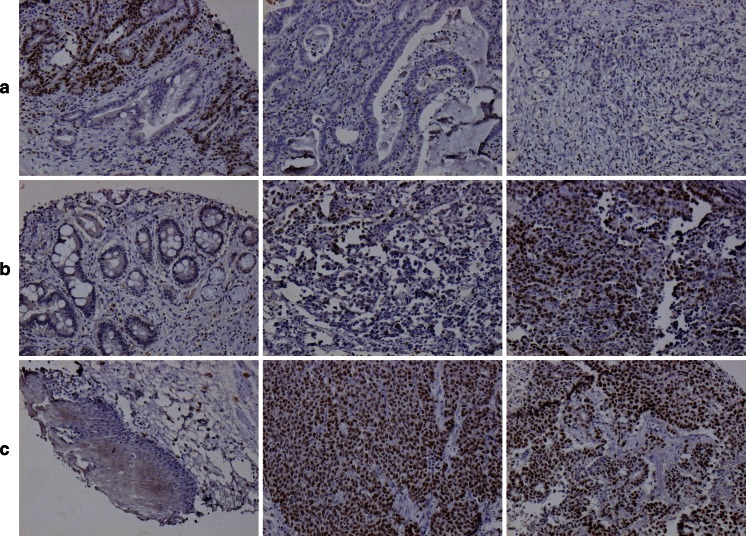
Examples of immunohistochemical SATB1 staining. Images (×10 magnification) of SATB1 expression in different tissue entities from three cases. *From left to right* (**a**) low- and high-grade dysplastic intestinal metaplasia (score 4), cancer (score 0), and metastasis (score 0 but with SATB1-positive lymphocytes) in a T2N2M0 esophageal cancer; (**b**) intestinal metaplasia (score 1), cancer (score 4), and metastasis (score 9) in a T3N1M0 cardiac cancer; (**c**) normal squamous epithelium (score 0), cancer (score 12), and metastasis (score 12) in a T3N3M0 cardiac cancer

SATB1 could be evaluated in 71/96 (74 %) samples with normal squamous epithelium, 125/131 (95 %) samples with normal gastric mucosa, 63/73 (86 %) samples with IM, 170/175 (97 %) primary tumors, and 79/81 (98 %) metastases. Immunohistochemical images are shown in Fig. 2.

**Fig. 3:**
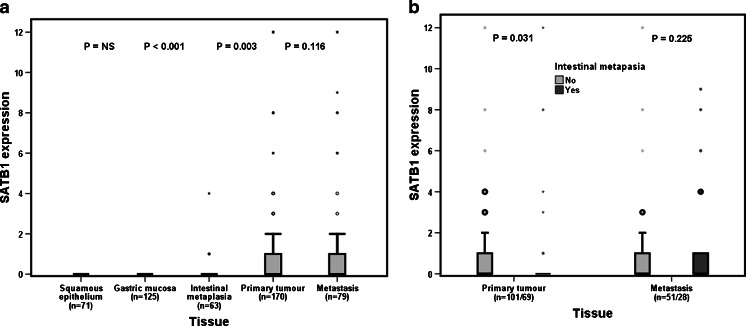
Visualization of SATB1 expression according to tissue type. **a** SATB1 expression according to tissue type in the entire cohort. **b** SATB1 expression in primary tumors (*left*) and metastases (*right*) with and without the presence of intestinal metaplasia (Barrett’s esophagus included)

As demonstrated in Fig. 3a, SATB1 expression was significantly higher in primary tumors (*n* = 53/170, 31.2 %) and metastases (*n* = 32/79, 40.5 %) than in normal squamous epithelium (*n* = 0/96, 0 %) and normal gastric mucosa (*n* = 0/131, 0 %) where no expression was seen. SATB1 expression did not differ between the primary tumors and metastases (*p* = 0.116). SATB1 expression was significantly higher in IM than that in normal tissue (*p* = 0.003), but the number of SATB1-expressing IM samples was very small (*n* = 8). Figure 3b shows that the expression of SATB1 was significantly lower in primary tumors with tumor-associated IM than that in primary tumors without tumor-associated IM (*p* = 0.031), but this difference was not maintained in metastases.

### Correlations of SATB1 expression in primary tumors with clinicopathological parameters

Table 1 lists the distribution of clinicopathological and investigative parameters according to SATB1 expression. Significant associations of SATB1 expression were found with younger age (*p* = 0.045) and more advanced N stage (*p* = 0.010). SATB1 expression was also significantly more common in esophageal compared to cardiac or gastric cancer (*p* = 0.033).

**Table 1:** Distribution of clinicopathological characteristics according to SATB1 expression

	SATB1 expression		
Factor, *n* (%)	Negative, 117 (68.9)	Positive, 53 (31.2)	*p* value
Age			0.045
Mean	71.4	67.9	
Median (range)	72.9 (42.6–94.4)	65.6 (48.2–87.2)	
Sex			0.492
Women	30 (25.6)	11 (20.8)	
Men	87 (74.4)	42 (79.2)	
T stage			0.243
1	11 (9.6)	7 (13.2)	
2	28 (24.6)	4 (7.5)	
3	58 (50.9)	32 (60.4)	
4	17 (14.9)	10 (18.9)	
Unknown	3	0	
N stage			0.010
0	47 (40.2)	9 (17.0)	
1	18 (15.4)	12 (22.6)	
2	26 (22.2)	14 (26.4)	
3	26 (22.2)	18 (34.0)	
Unknown	0	0	
M stage			0.288
0	93 (86.9)	40 (93.0)	
1	14 (13.1)	3 (7.0)	
Unknown	10	10	
Differentiation grade			0.853
High	3 (3.0)	3 (6.4)	
Intermediate	28 (28.3)	11 (23.4)	
Low	68 (68.7)	33 (70.2)	
Unknown	18	6	
Adjuvant Radio/Chemotherapy			0.376
No	105 (92.9)	43 (87.8)	
Yes, with oxaliplatin	1 (0.9)	1 (2.0)	
Yes, without oxaliplatin	4 (3.5)	4 (8.2)	
Yes, NOS	3 (2.7)	1 (2.0)	
Unknown	4	4	
Location			0.033
Esophageal	33 (29.2)	26 (49.1)	
Cardiac	34 (30.1)	11 (20.8)	
Gastric	46 (40.7)	16 (30.2)	
Unknown	4	0	
Ki67 expression			0.201
0–20 %	43 (37.1)	13 (24.5)	
21–50 %	33 (28.4)	19 (35.8)	
>50 %	40 (34.5)	21 (39.6)	
p53 expression			0.059
0–1 %	37 (31.6)	11 (20.8)	
2–50 %	30 (25.6)	11 (20.8)	
>50 %	50 (42.7)	31 (58.5)	
Missing	1	0	
SATB2 expression			<0.001
SATB2-negative	109 (93.2)	38 (71.7)	
SATB2-positive	8 (6.8)	15 (28.3)	
Resection margin			0.271
R0	82 (70.1)	37 (69.8)	
R1	27 (23.1)	6 (11.3)	
R2	8 (6.8)	10 (18.9)	

### Impact of SATB1 expression on survival

**Fig. 4:**
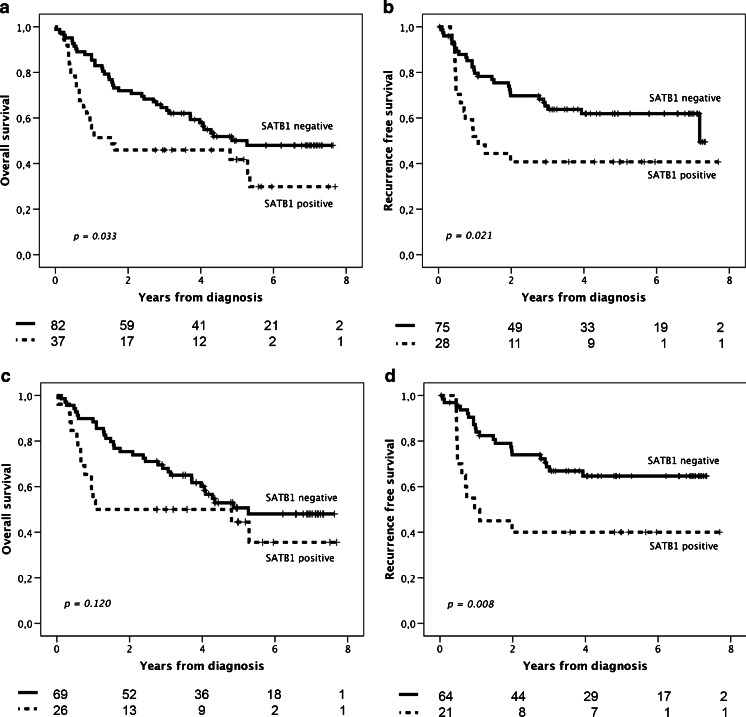
Kaplan-Meier estimates of survival and recurrence according to SATB1 expression. Overall survival (**a**) and recurrence-free survival (**b**), according to SATB1 expression in patients with radically resected tumors. Overall survival (**c**) and recurrence-free survival (**d**) in patients with radically resected tumors and distant metastasis-free disease

Kaplan-Meier analysis of radically resected (R0) tumors revealed both decreased OS (*p* = 0.033) and shorter RFS (*p* = 0.021) for patients with SATB1-positive compared to SATB1-negative tumors (Fig. 4). SATB1expression was significantly associated with shorter RFS in patients with a R0 tumor and distant metastasis-free (M0) disease (*p* = 0.008) but not with OS.

As demonstrated in Table 2, the prognostic value of SATB1 was confirmed in unadjusted Cox regression analysis for OS in patients with R0 resection (HR = 1.74; 95 % CI 1.04–2.90, *p* = 0.036) and for RFS in patients with R0 resection/M0 disease (HR = 2.53; 95 % CI 1.24–5.16, *p* = 0.011). These associations remained significant in adjusted analysis (HR = 2.30; 95 % CI 1.32–4.01, *p* = 0.003 for OS and HR = 3.88; 95 % CI 1.72–8.72, *p* = 0.001 for RFS), and also when tumor location was included in the adjusted model (data not shown).

**Table 2:** Impact of SATB1 expression on relative risks of death in cases with radically resected primary tumors and risk of recurrence in patients with radically resected primary tumors and distant metastasis-free disease

	Overall survival—R0 resection					Recurrence-free survival time—R0 resection + M0 disease				
	*n* (events)	Unadjusted	*p* value	Adjusted	*p* value	*n* (events)	Unadjusted	*p* value	Adjusted	*p* value
		HR (95 % CI)		HR (95 % CI)			HR (95 % CI)		HR (95 % CI)	
Age										
Continuous	119 (63)	1.05 (1.02–1.07)	<0.001	1.08 (1.05–1.10)	<0.001	86 (33)	1.00 (0.97–1.03)	0.940	1.05 (1.02–1.09)	0.004
Gender										
Female	26 (15)	1.00		1.00		16 (3)	1.00		1.00	
Male	93 (48)	0.82 (0.46–1.46)	0.496	0.96 (0.49–1.85)	0.893	69 (30)	2.65 (0.81–8.69)	0.108	3.57 (0.99–12.87)	0.052
T stage										
T1	18 (5)	1.00		1.00		11 (1)	1.00		1.00	
T2	31 (17)	2.36 (0.87–6.42)	0.091	1.61 (0.53–4.87)	0.399	24 (7)	3.65 (0.45–29.64)	0.226	6.66 (0.51–86.07)	0.146
T3	54 (32)	2.67 (1.04–6.86)	0.042	1.15 (0.40–3.31)	0.791	41 (20)	6.72 (0.90–50.10)	0.063	7.08 (0.65–77.35)	0.109
T4	15 (9)	3.25 (1.08–9.71)	0.035	1.27 (0.37–4.37)	0.705	8 (5)	11.19 (1.30–96.22)	0.028	5.43 (0.41–71.32)	0.198
N stage										
N0	45 (17)	1.00		1.00		35 (2)	1.00		1.00	
N1	23 (11)	1.41 (0.66–3.01)	0.376	1.87 (0.85–4.11)	0.118	18 (10)	12.44 (2.72–56.86)	0.001	20.12 (4.10–98.77)	<0.001
N2	27 (17)	2.14 (1.09–4.20)	0.027	3.32 (1.65–6.70)	0.001	22 (13)	16.26 (3.66–72.26)	<0.001	24.68 (5.34–113.93)	<0.001
N3	24 (18)	3.61 (1.84–7.07)	<0.001	5.00 (2.43–10.30)	<0.001	10 (8)	27.79 (5.84–132.32)	<0.001	64.58 (12.21–341.67)	<0.001
M stage										
M0	95 (48)	1.00		1.00			1.00		1.00	
M1	10 (9)	2.85 (1.39–5.84)	0.004	1.66 (0.72–3.82)	0.235		–		–	
Differentiation										
High-moderate	36 (19)	1.00		1.00		30 (9)	1.00		1.00	
Low	65 (39)	1.12 (0.65–1.95)	0.676	1.23 (0.70–2.16)	0.475	38 (19)	1.77 (0.80–3.93)	0.157	2.50 (1.07–5.85)	0.034
SATB1 expression										
Negative	82 (40)	1.00		1.00		64 (21)	1.00		1.00	
Positive	37 (23)	1.74 (1.04–2.90)	0.036	2.30 (1.32–4.01)	0.003	21 (12)	2.53 (1.24–5.16)	0.011	3.88 (1.72–8.72)	0.001

In the entire cohort, SATB1 positivity was not significantly associated with overall survival but with a significantly shorter RFS in unadjusted (HR = 1.68; 95 % CI 1.04–2.71, *p* = 0.032) but not in adjusted analysis (data not shown).

As shown in Supplemental Fig. 1, SATB2 was expressed to a very limited extent in the examined tissues. No correlation with RFS or OS was seen (data not shown), and hence, no further statistical analyses were performed. Expression of Ki67 and p53 in primary tumors and metastases had no prognostic significance (data not shown). Kaplan-Meier analysis revealed a trend toward longer OS for patients with tumor-adjacent IM compared with patients without tumor-adjacent IM (*p* = 0.054, data not shown). Patients with R0 tumors had a prolonged OS compared with patients with non-R0 tumors (*p* = <0.001, data not shown).

## Discussion

We have examined the expression of SATB1 in matched normal squamous epithelium, normal gastric mucosa, Barrett’s esophagus, gastric intestinal metaplasia, and primary and metastatic adenocarcinoma in patients with cancer of the upper gastrointestinal tract with known clinical outcome. We show that SATB1 expression in primary tumors is an independent prognostic marker for shorter OS and shorter RFS in patients with radically resected tumors. These results are in line with several previous studies indicating that SATB1 expression correlates with a more aggressive phenotype and worse prognosis in several types of cancer [9, 12–16].

In our cohort, involvement of resection margins was significantly associated with poor prognosis. This not only validates the use of the cohort for biomarker studies but also strengthens the prognostic value of SATB1 expression in radically resected tumors, in particular since an earlier study was limited by a lack of information on residual tumor after surgery [13]. SATB1 expression was prognostic for both OS and RFS in R0 tumors only when all R0 tumors were included. When R0 tumors with distant metastasis (M1) were excluded, SATB1 was only significantly associated with OS, but this might change with a longer follow-up time. SATB1 expression was not different between primary tumors and metastases, indicating that it is sufficient to examine only the primary tumor for prognostic purposes.

We evaluated SATB1 expression using an approach similar to that used in a previous study [22]. As in previous studies, we found that lymphocytes are suitable as internal positive control [9, 18]. We also confirm that expression of SATB1in a limited fraction of the tumor cells already confers poor prognosis [12].

The prognostic value of SATB1 is controversial in different tumor types, which may be due to tissue-dependent regulatory functions of SATB1 [18]. Possible implications of differences in methodologies and materials also need to be considered, notably the possibility of discordance between messenger RNA (mRNA) and protein levels [29]. Immunohistochemistry (IHC) allows assessment of protein expression of a putative biomarker in specific cell types and even its subcellular location. Our results are in line with those reported by Han et al., in that expression of SATB1 protein independently predicts worse outcome [12]. Studies on SATB1 mRNA levels failed to demonstrate independent prognostic value for SATB1 expression [19, 20]. Such mRNA studies usually include both tumor and normal cells, which is an inherent source of error. For biomarker studies therefore, IHC is a more reliable method of investigation. The use of different antibodies is another potential source of controversy. SATB1 is highly homologous to SATB2 and the specificity of SATB1 and SATB2 antibodies has been questioned in previous studies [18]. The specificity of antibodies for SATB1 and SATB2 needs to be thoroughly validated [9], as we have performed in this study. This is all the more important as several studies have indicated that SATB1 and SATB2 have antagonistic qualities [9, 14, 30]. As an example, SATB2 expression was a favorable prognostic marker in colorectal cancer [30, 31].

SATB1 remained significant as a prognostic factor when tumor location was included in the adjusted Cox regression model, indicating that SATB1 is prognostic in upper gastrointestinal tract cancer regardless of its anatomical location. Interestingly, SATB1 expression was significantly lower in primary tumors associated with IM than in primary tumors not associated with IM. This is in line with at least two different pathways of gastroesophageal carcinogenesis, one intestinal (arising from dysplasia in IM) and one non-intestinal (arising from cardia-type mucosa), the former being associated with better overall survival [32]. Our cohort showed a similar trend, which is in agreement with SATB1 expression as a negative prognostic factor.

ERBB2 (HER2) is an important drug target in breast cancer and an increasingly important target in gastric cancer [8]. SATB1 upregulates ERBB2 (HER2) expression [9, 12], which draws attention to SATB1 as a candidate drug target. Knockdown of SATB1 in aggressive breast cancer cell lines caused complete reversal of tumor growth and metastatic abilities in vivo and introduction of SATB1 decoy DNA drastically reduced invasive and metastatic capacity of SATB1-positive cell lines [33]. Similar results were reported in colorectal cancer [9, 12, 16]. SATB1 downregulates expression of E-cadherin, which is a characteristic event in epithelial to mesenchymal transition and an important step in invasion and metastasis [34, 35].

Studies, using the same well-validated anti-SATB1 antibody as in our study, have indicated that SATB1 contributes to chemotherapy multidrug resistance [24], which provides additional arguments in favor of SATB1-blocking as a novel therapeutic approach.

None of the patients in this study had received neoadjuvant treatment. This rules out any possibility that biomarker expression was affected by treatment, which must be considered a strength of this study. A further strength is that all available surgically treated tumors were included consecutively, which excludes risk of selection bias. A limitation of the present study is the use of TMAs with a risk of sampling bias. Our TMA design limits this as duplicate cores were taken from different blocks of the primary tumor and different lymph node metastases in cases with more than one metastasis. Furthermore, even with full-face sections, sampling bias is not excluded as these also represent only a limited fraction of the tumor. An advantage of the TMA approach is the high number of tumors that can be studied, which conceivably might compensate for false negative or positive tissue cores [36].

In conclusion, we show that SATB1 is an independent prognostic biomarker in patients with radically resected adenocarcinomas of the upper gastrointestinal tract.

## Electronic supplementary material

Below is the link to the electronic supplementary material.

ESM 1 (DOCX 95 kb)

Supplemental Figure 1 Visualization of SATB2 expression according to tissue type. (A) SATB2 expression according to tissue type in the entire cohort. (B) SATB2 expression in primary tumours (left) and metastases (right) with and without presence of intestinal metaplasia (Barrett’s esophagus included). (JPEG 130 kb)
