# Supplementary material for: SATB1 is an independent prognostic factor in radically resected upper gastrointestinal tract adenocarcinoma
Source: Virchows Arch. 2014 Oct 19;465(6):649–59. doi: 10.1007/s00428-014-1667-6 (PMC4245492; doi:10.1007/s00428-014-1667-6)
Supplement: Supplementary file 1 — (DOCX 95 kb) [file 428_2014_1667_MOESM1_ESM.docx]

**Supplemental Table 1 – Patient and tumour characteristics in the cohort**

| **Factor** | **Entire cohort**  **(n=175)**  **n (%)** | **Esophagus**  **(n=60)**  **n (%)** | **Cardia**  **(n=45)**  **n (%)** | **Stomach**  **(n=66)**  **n (%)** | *P* |
| --- | --- | --- | --- | --- | --- |
| **Age**  Mean  Median  (Range) | 70.2  69.8  42.6-94.4 | 76.9  66.02  48.2-88.5 | 69.9  68.7  48.7-88.5 | 72.0  72.6  42.6-94.4 | 0.080 |
| **Sex**  Women  Men | 41 (23.4)  134 (76.6) | 6 (10.0)  54 (90.0) | 12 (26.7)  33 (73.3) | 20 (30.3)  46 (69.7) | 0.007 |
| **T stage**  1  2  3  4  Unknown | 19 (11.0)  32 (18.6)  94 (54.7)  27 (15.7)  3 | 9 (15.3)  10 (16.9)  34 (57.6)  6 (10.2)  1 | 3 (6.8)  4 (9.1)  33 (75.0)  4 (9.1)  1 | 6 (9.2)  17 (26.2)  26 (40.0)  16 (24.6)  1 | 0.265 |
| **Resection margins**  R0  R1  R2 | 122 (69.7)  34 (19.4)  19 (10.9) | 38 (63.3)  10 (16.7)  12 (20.0) | 30 (66.7)  11 (24.4)  4 (8.9) | 51 (77.3)  12 (18.2)  3 (4.5) | 0.016 |
| **Examined nodes**  Mean  Median  Range  Unknown | 29.0  30.2  1-112  14 | 36.6  33.5  10-72  2 | 29.7  28.00  8-48  1 | 25.8  23.0  1-112  11 | <0.001 |
| **N stage**  0  1  2  3 | 59 (33.7)  30 (17.1)  41 (23.4)  45 (25.7) | 15 (25.0)  11 (18.3)  15 (25.0)  19 (31.7) | 12 (26.7)  7 (15.6)  14 (31.1)  12 (26.7) | 28 (42.4)  12 (18.2)  12 (18.2)  14 (21.2) | 0.032 |
| **M stage**  0  1  Unknown | 137 (88.4)  18 (11.6)  20 | 51 (86.4)  8 (13.6)  1 | 40 (88.9)  5 (11.1) | 45 (91.8)  4 (8.2)  19 | 0.377 |
| **Differentiation grade**  High  Intermediate  Low  Unknown | 6 (4.0)  40 (26.8)  103 (69.1)  26 | 3 (5.9)  21 (41.2)  27 (52.9)  9 | 1 (2.5)  9 (22.5)  30 (75.0)  5 | 1 (1.8)  9 (16.4)  45 (81.8)  11 | 0.002 |
| **Adjuvant Radio/**  **Chemotherapy**  No  RT  CT with oxaliplatin  CT without oxaliplatin  RT + CT without oxaliplatin  RT + CT, NOS  Yes, NOS  Unknown | 150 (85.7))  1 (0.6)  2 (1.1)  3 (1.7)  6 (3.4)  2  3 (1.7)  8 | 54 (93.1)  1  0  0  2 (3.4)  0  1 (1.7)  2 | 39 (90.7)  0  0  2 (4.7)  2 (4.7)  0  0  2 | 55 (85.9)  0  2 (3.1)  1 (1.6)  2 (3.1)  2 (3.1)  2 (3.1)  2 | 0.196 |
| **Location**  Esophagus  GE-junction  Stomach  Unknown | 60 (35.1)  45 (26.3)  66 (38.6)  4 | -  -  - | -  -  - | -  -  - |  |
| **Follow-up**  Mean  Median  Range | 2.92  2.27  0.01-7.70 | 2.97  2.65  0.26-7.70 | 2.87  2.17  0.01-7.64 | 2.92  2.15  0.03-7.60 | 0.848 |
| **Vital status**  Alive  Dead | 64 (36.6)  111 (63.4) | 27 (45.0)  33 (55.0) | 14 (31.1)  31 (68.9) | 22 (33.3)  44 (66.7) | 0.184 |
| **Recurrence**  No  Yes  Unknown | 64 (46.4)  74 (53.6)  37 | 24 (46.2)  28 (53.8)  8 | 14 (38.9)  22 (61.1)  9 | 25 (50.0)  25 (50.0)  16 | 0.705 |
