# Supplementary figures and images for: SATB1 is an independent prognostic factor in radically resected upper gastrointestinal tract adenocarcinoma
Source: Virchows Arch. 2014 Oct 19;465(6):649–59. doi: 10.1007/s00428-014-1667-6 (PMC4245492; doi:10.1007/s00428-014-1667-6)

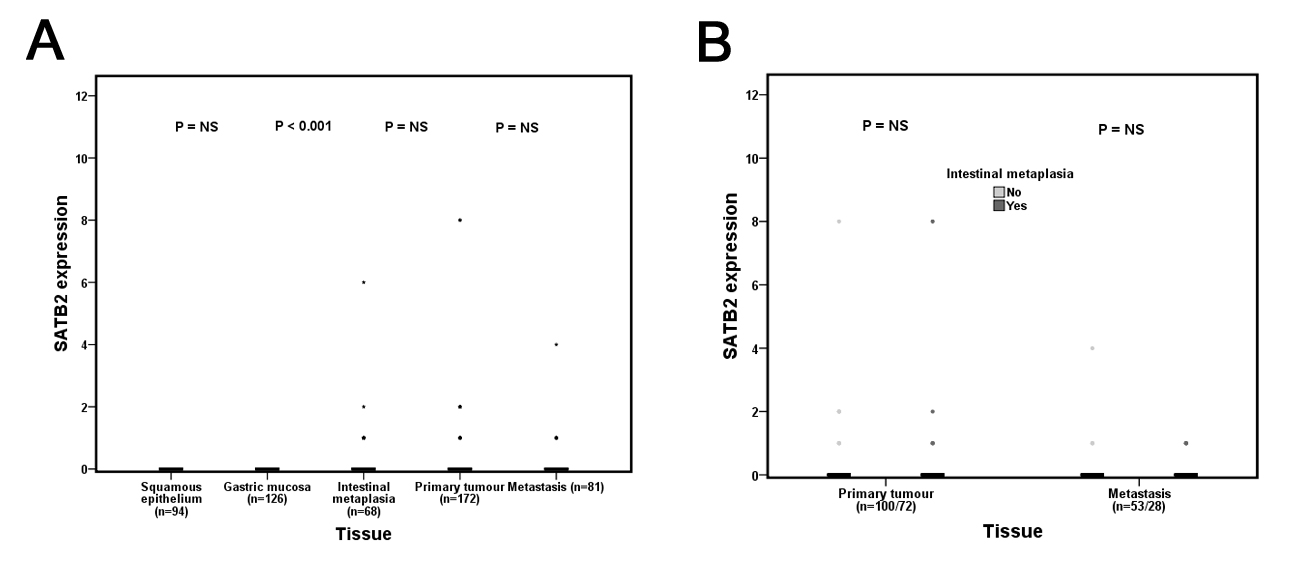

Supplement: Supplementary file 2 — Visualization of SATB2 expression according to tissue type. (A) SATB2 expression according to tissue type in the entire cohort. (B) SATB2 expression in primary tumours (left) and metastases (right) with and without presence of intestinal metaplasia (Barrett’s esophagus included). (JPEG 130 kb) [file 428_2014_1667_Fig5_ESM.jpg]
